# Adaptation in *Bacillus cereus*: From Stress to Disease

**DOI:** 10.3389/fmicb.2016.01550

**Published:** 2016-10-04

**Authors:** Catherine Duport, Michel Jobin, Philippe Schmitt

**Affiliations:** Sécurité et Qualité des Produits d’Origine Végétale, UMR0408, Avignon Université, Institut National de la Recherche AgronomiqueAvignon, France

**Keywords:** *Bacillus cereus*, redox homeostasis, oxygen sensing, acidic pH, metabolism

## Abstract

*Bacillus cereus* is a food-borne pathogen that causes diarrheal disease in humans. After ingestion, *B. cereus* experiences in the human gastro-intestinal tract abiotic physical variables encountered in food, such as acidic pH in the stomach and changing oxygen conditions in the human intestine. *B. cereus* responds to environmental changing conditions (stress) by reversibly adjusting its physiology to maximize resource utilization while maintaining structural and genetic integrity by repairing and minimizing damage to cellular infrastructure. As reviewed in this article, *B. cereus* adapts to acidic pH and changing oxygen conditions through diverse regulatory mechanisms and then exploits its metabolic flexibility to grow and produce enterotoxins. We then focus on the intricate link between metabolism, redox homeostasis, and enterotoxins, which are recognized as important contributors of food-borne disease.

## Introduction

Micro-organisms display astonishing abilities to survive and grow in hostile environments. Some have adapted their life cycles to extreme conditions (thermophiles, psychrophiles, halophiles, acidophiles, etc.), but all can marshal temporary adaptation mechanisms to help them survive until conditions have become more favorable. When environmental conditions change, a cell must modify its physiology accordingly to cope with them and survive or reproduce. However, adaptation has limits, which depend on the microorganism and on the environmental variables that have become extreme, upset the cell’s equilibrium, and caused stress. Limits to adaptation depend on the micro-organism’s intrinsic capacity to cope; the physiological responses the cell can call on to address environmental variations include changes in metabolism and/or mechanisms of adaptation and resistance.

*Bacillus cereus* is a Gram-positive, facultative anaerobe, rod-shaped endospore-forming bacterium that is known to inhabit primarily the soil, where it can complete its saprophytic life cycle (Vilain et al., 2006). As a result of its saprophytic soil life cycle, *B. cereus* is found in water, vegetables, and many other food ingredients, resulting in the contamination of a wide variety of finished food products. Ingestion of contaminated foods by humans can lead to two types of gastrointestinal infections, both damaging the host epithelium. The emetic type of food poisoning is caused by the ingestion of food containing the toxin cereulide, whereas the diarrheal type depends on the ingestion of *B. cereus* cells followed by the production of virulence factors in the human small intestine (Stenfors Arnesen et al., 2008; Senesi and Ghelardi, 2010; Ceuppens et al., 2012). *B. cereus* cells originate from ingested vegetative cells that survive gastric passage (Wijnands et al., 2009) and/or from ingested spores which first adhere to the intestinal mucosa and then germinate. Recent evidence suggests that *B. cereus*-induced diarrhea is not caused by massive *B. cereu*s proliferation and virulence factor production in the intestinal lumen but by localized growth and virulence factor production at the hosts mucus layer or epithelial surface (Ceuppens et al., 2012). These virulence factors then damage the nearby epithelial cells by pore formation, resulting in microvilli damage and osmotic lysis of the host’s epithelial cells and eventually diarrhea (Beecher et al., 1995; Hardy et al., 2001; Minnaard et al., 2001; Lindback et al., 2004; Ramarao and Lereclus, 2006; Fagerlund et al., 2008). In the vicinity of the epithelial layer, *B. cereus* is exposed to different oxygen concentrations and different oxidoreduction potentials (ORP; Moriarty-Craige and Jones, 2004; Marteyn et al., 2010a,b) that induce compensatory metabolic pathways in an attempt to maintain the intracellular redox state. The cellular redox status governs the status of redox-sensitive macromolecules and protects against endogenous oxidative stress. Recent studies suggested that virulence factor production by *B. cereus* is dynamic and shaped by cellular oxidation (Madeira et al., 2015). Thus, there is an intricate link between metabolism, redox homeostasis, and virulence factor production. In the first part of this review, we focus on how microorganisms and *B. cereus* detect and respond to acid stress, and review the different behavioral, physiological and molecular mechanisms underpinning acid stress adaptation. In the second part, we will begin by describing the basics of *B. cereus* physiology and will then discuss how metabolism and redox global regulators influence the production of virulence factors under changing oxygen conditions.

## Acid Stress Resistance

Acid resistance is especially important for *B. cereus* that must survive the acidic pH of the stomach – which is 1.5 in the fasting state (Van de Guchte et al., 2002) and rises to 3–5 after ingestion of food (Cotter and Hill, 2003) – before entering and colonizing the small intestines or colon (Stenfors Arnesen et al., 2008). Acid stress is also frequently encountered naturally in many foods, as a result of the use of weak organic acids or short-chain (volatile) fatty acids (FA; e.g., acetic acid, citric acid, and propionic acids) as food preservatives (Alvarez-Ordonez et al., 2010a). Thus, the ability to adapt to an acidified environment is crucial to the virulence of a food-borne pathogen such as *B. cereus.*

Neutrophilic bacteria have evolved multiple tolerance or resistance mechanisms to prevent cell damage due to acid stress; these are generally referred to as acid tolerance responses (ATRs) and acid resistance mechanisms, respectively. Which system(s) plays the dominant role(s) depends on: (i) the phase of growth of the cells when the ATR is elicited [exponential phase- versus stationary phase- ATR]; and/or (ii) whether certain amino acids are present during exposure to the acidic pH; and/or (iii) whether acidification of the environment results from inorganic or organic acids (Van de Guchte et al., 2002; Alvarez-Ordonez et al., 2010b). *B. cereus* vegetative cells, like many other bacteria, are able to induce an ATR (Jobin et al., 2002; Thomassin et al., 2006). In addition, it has been shown that *B. cereus* cells pre-adapted at pH 6.3 coped better with both ethanol stress (12%) and heat stress (49°C) (Browne and Dowds, 2002), suggesting that ATR and/or induction of acid-resistance mechanisms confer cross-protection for other stresses. In this article, we review some model acid-resistant and their related acid resistance mechanisms to understand how *B. cereus* can adapt to acid stress.

### Common Mechanisms of Acid Resistance

**FIGURE 1:**
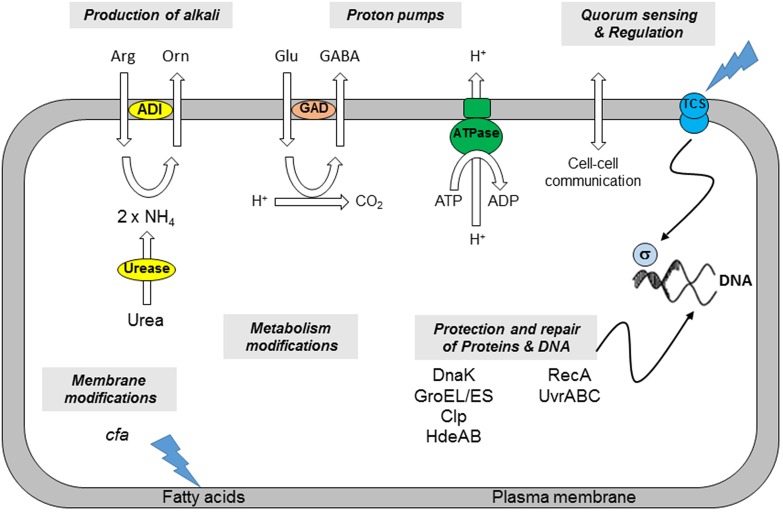
**Mechanisms involved in *B. cereus* acid resistance (adapted from Cotter and Hill, 2003).** (i) The production of alkali by the ADI or urease system increases the internal pH of the cell. (ii) Proton pumps such as the F_1_F_0_-ATPase or that utilized by the GAD system bring about an increase in pHi. (iii) Cell density affects cell-to-cell communication. The involvement of Two Component Systems (TCS) and sigma factors can induce minor or global responses. (iv) Chaperones, proteases, and heat shock proteins (HdeAB) protect cellular proteins or degrade them if damaged. (v) DNA damaged as a consequence of a low pHi can be repaired through the excision of errors or the restarting of stalled replication forks. (vi) Modifications of membrane fatty acids composition affects the properties of the plasma membrane. (vii) Metabolism of the cells may be modified in response to environmental changes.

Micro-organisms deploy various mechanisms and strategies to address the hostile conditions of low-pH environment, e.g., modification of the architecture and composition of the membrane, change in metabolism and production of alkaline substances, and homeostasis of internal pH (**Figure 1**).

#### Modification of the Membrane

The cell membrane of bacteria is in direct contact with external media. It is therefore the first one to be affected by harmful environmental conditions, e.g., an acidic medium. The fluidity of the membrane is important for cells, as it can affect membrane functions such as biochemical reactions, transport systems, and protein secretion. The membrane FA composition is responsible for the maintenance of membrane fluidity, and a number of studies have suggested a relationship between membrane fluidity and stress adaptation. Acid adaptation generally decreased membrane fluidity, and this is likely linked to the overall increase in short-chain saturated FA as observed in *Escherichia coli. Salmonella*, and *Listeria* (Kwon and Ricke, 1998a,b; Yuk and Marshall, 2004; Moorman et al., 2008; Alonso-Hernando et al., 2010). However, in some oral bacteria, exposure to acidic condition resulted in increased level of long-chain monounsaturated FA, and fluidity (Fozo and Quivey, 2004; Fozo et al., 2004; Papadimitriou et al., 2007). In *E. coli*, acid adaptation causes the conversion of a significant proportion of the unsaturated FA to their cyclic derivatives, known as cyclopropane fatty acids (CFA) during the transition from exponential to stationary phase (Merrell and Camilli, 2002; Merrell et al., 2002). The CFAs are formed by CFA synthase, which is encoded by *cfa*. Defective *cfa* mutants are unable to produce CFA and are sensitive to low pH (Booth, 2002). Phospholipid composition is also important in *E. coli*: stationary phase *E. coli* cells are much more sensitive to acid shock at pH 3 in the absence of the main phospholipid, phosphatidylethanolamine (Canet et al., 2003). Membrane adaptation in response to acid stress has not been yet investigated in *B. cereus*. However, it has been shown that *B. cereus* cells increase membrane fluidity by altering membrane FA composition in response to cold and saline stresses (Ultee et al., 2000; De Sarrau et al., 2012).

#### Production of Alkali

Some bacteria produce alkaline compounds, and specifically ammonia, to neutralize internal pH when exposed to an acidic environment. Ammonia is generated by two systems, the urease and the arginine deiminase systems (ADI; Van de Guchte et al., 2002; Cotter and Hill, 2003). In the urease system, urea is hydrolysed to two molecules of ammonia and one of CO_2_ by urease. In the arginine deiminase pathway, arginine is catabolized to ornithine with the release of ammonia and CO_2_. The urease system is more widespread and can protect some oral bacteria against acid-induced changes (Cotter and Hill, 2003; Wilson et al., 2014). Although the catalytic reaction is relatively simple, biogenesis of a functional urease is a highly complex process requiring at least seven genes, which are generally organized in operons [*ureABCEFGD*, (Marquis and Hager, 2000)]. The expression of bacterial ureases can be constitutive, but more often it is regulated by environmental conditions. Commonly, in enteric bacteria, the presence of urea or limitation for nitrogen can induce urease gene transcription, generally through activation of transcription. Specifically, urease expression is almost completely repressed at neutral pH values, regardless of the limiting nutrient or growth rate. In acidic conditions, the urease genes become rapidly derepressed, and expression then becomes sensitive to carbohydrate availability and rate of growth, with the highest levels of expression under conditions of carbohydrate excess and fast growth rate (Chen and Burne, 1996; Chen et al., 1996; Cotter and Hill, 2003). *B. cereus* is in contact with the urea present in its diverse habitats, such as soil, human urine, human saliva (2.3–4.1 mM) (Mobley, 2000; Dawes and Dibdin, 2001), stomach (4.8 mM) (Neithercut et al., 1993), blood (1.7–8.3 mM) (Mackay and Mackay, 1927), and some animal foods (e.g., milk), which contain 4.4–6.4 mM urea (Carlsson et al., 1995). Although the activity of urease can play an important role in the life cycle of *B. cereus*, little information is available on its role in nitrogen metabolism and in acid stress survival in this bacterium. The ADI has also been identified in a broad variety of bacteria, including *B. cereus* (Van de Guchte et al., 2002; Cotter and Hill, 2003; Senouci-Rezkallah et al., 2011). This system is a three-enzyme pathway that initially converts arginine to citrulline and ammonia via arginine deiminase (encoded by *arcA*). The citrulline is then transformed into ornithine and carbamyl phosphate by ornithine transcarbamylase (encoded by *arcB*). The third enzyme in the pathway, carbamate kinase (encoded by *arcC*), cleaves carbamyl phosphate to ammonia and CO_2_, concomitantly donating the phosphate to ADP to produce ATP (Griswold et al., 2004). Similar to ureolysis, the net reaction yields two molecules of ammonia and one of CO_2_, but also provides ATP for growth. Thus, many ADI-positive bacteria can grow with arginine as the sole source of energy. ADI-positive organisms often coordinately regulate the synthesis of an arginine:ornithine antiporter (encoded by *arcD*) (Cotter and Hill, 2003; Budin-Verneuil et al., 2006). The expression of *arcABC* operon is induced by low pH or arginine and is suppressed by excess oxygen pressure in *Listeria monocytogenes* (Ryan et al., 2009). The *arcABC* operon also contributes to the growth and survival of *Lactobacillus plantarum* in low-pH environment (De Angelis et al., 2002; Spano et al., 2004). In *B. cereus*, arginine deiminase gene *arcA* showed significant up-regulation upon exposure to non-lethal acid shock at pH 5.4–5.5 (Mols et al., 2010a; Senouci-Rezkallah et al., 2011), suggesting that ADI may be of great importance for *B. cereus* survival in low pH environments.

#### Homeostasis of Internal pH

Internal pH (pHi) is an important factor in bacterial physiology, and cells regulate its value precisely. The regulation of pHi implies a heightened control of membrane permeability to protons, which can take place *via* the ion transporters that facilitate proton entry. In general, growth studies have shown that pH_i_ falls with culture pH (Russell, 1991; O’Sullivan and Condon, 1999; Siegumfeldt et al., 2000). In *B. cereus*, the pH_i_ in continuously cultured cells sampled at equilibrium fell with growth rate, showing that growth rate has an effect on pH_i_ (Thomassin et al., 2006). pHi is disturbed by two main factors: (i) passive movement of protons through the cytoplasmic membrane, and (ii) the production of acids and/or bases in the cytoplasm. Thus, in fermenting bacteria, the accumulation of acidic fermentation products in the cytoplasm can decrease pH_i_, despite continuous efflux of protons (Russell and Diez-Gonzalez, 1998). The organic acids present in the culture medium can also impede pH_i_ homeostasis, especially when pH_i_ > external pH (pHe). Bacteria that can allow their pHi to decrease, so as to keep a low ΔpH (pH_i_ – pH_e_), may be more acid-resistant than those that keep their pH_i_ neutral (Siegumfeldt et al., 2000). Non-dissociated organic acids freely cross the permeable membrane lipid bilayer, limiting their accumulation in the cell. In bacteria that maintain a higher pH_i_, organic acids dissociate in the more alkaline cytoplasm, and accumulate in the cell, halting growth (Mercade et al., 2000). Their dissociation depends on their p*K*a value, generally less than 5.0. The accumulation of these organic acids in anionic form depends on the pH gradient across the membrane (Russell, 1991). To keep pH_i_ at a value that will conserve the cell’s physiological integrity, bacteria can use many different strategies to control proton flux. These include (i) active transport of protons across the membrane (via the F1F0-ATPase activity), (ii) decarboxylation systems [glutamate decarboxylase (GAD), arginine decarboxylase (AD), and lysine decarboxylase], and (iii) the buffering ability of their cytoplasm.

##### F1F0-ATPase

**FIGURE 2:**
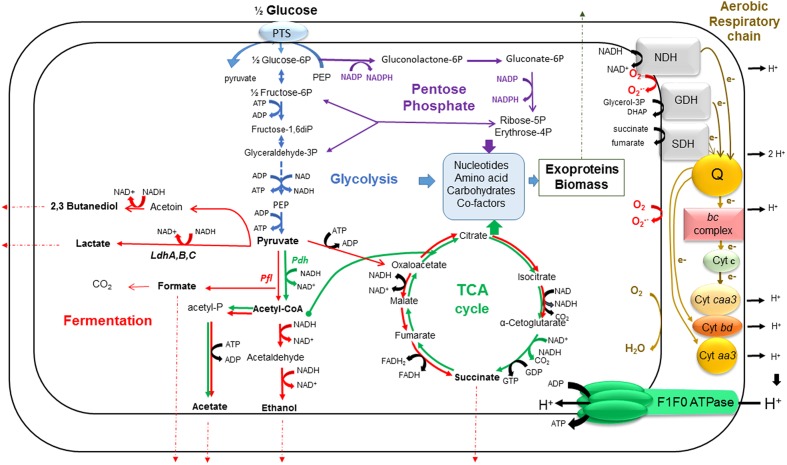
**A simplified view of *B. cereus* central metabolism.** This schematic shows our current understanding of how glycolysis (blue), the PPP (purple), the fermentation pathways (red), the TCA cycle (green) and aerobic respiratory chain (brown) are interconnected in growing *B. cereus* cells. The fermentation end products are shown in bold. The electron transport in the respiratory chain goes through dehydrogenation of NADH, as a first step. Electrons are transferred from NADH to menaquinone pool (Q), which serves as an electron carrier, and then, the pathway goes through two main branches. The first is one in which electrons go through the *bc* complex, cytochrome *c* and cytochrome oxidase *caa3*, in turn. The second contains only quinol oxidases (cytochromes *bd* or *aa3*). In each branch, electrons are finally accepted by oxygen molecules. The transfer of electrons is coupled to the formation of a proton gradient across the membrane which is tapped by F_1_F_0_-ATPase to generate ATP from ADP and Pi. ROS are generated as by-product from this process. Ldh, lactate dehydrogenase; Pdh, pyruvate dehydrogenase; Pfl, pyruvate formate lyase; NDH, NADH:menaquinone reductase; SDH, succinate:menaquinone oxidoreductase; GDH, glycerol-3-P:menaquinone reductase.

A number of studies have demonstrated a role for F_1_F_0_-H^+^ translocating ATPase in pH_i_ homeostasis (Miwa et al., 1997; Kullen and Klaenhammer, 1999; Cotter and Hill, 2003; Fortier et al., 2003). The F_0_F_1_-ATPase is a well-established mover of protons across the cell membrane (**Figure 2**). This complex couples the energy released as protons move into the cell to the generation of ATP from ADP and P_i._ The ATPase can also function in the opposite direction, hydrolyzing ATP to pump protons out of the cell. F_1_F_0_-ATPase is composed of two protein complexes (Negrin et al., 1980; Takemoto et al., 1981; Cotter and Hill, 2003). The F0 complex, which is integrated in the membrane, allows protons to cross the membrane. The F_1_ complex bears the catalytic site for the synthesis of ATP. The locus *atp* codes for the five sub-units α, β, δ, γ, and ε that form the complex F1, and the three subunits a, b and c that compose F_0_. It has been shown that the expression of *atp* was induced by an acid pH (Kullen and Klaenhammer, 1999; Quivey et al., 2001; Fortier et al., 2003). In *E. coli*, F_1_F_0_-ATPase acts as a proton pump during acid stress, driving protons out of the cell with a parallel hydrolysis of ATP (Richard and Foster, 2003). The F_1_F_0_-ATPase of *B. cereus* has been isolated from cytoplasmic membranes, purified and characterized. Structurally, *B. cereus* F_1_F_0_-ATPase resembles the enzyme isolated from *E. coli* and *B. subtilis* (Voelz, 1964; Banfalvi et al., 1980a,b). However, its enzymatic activity is insensitive to pH, and to *N,N’*-dicyclohexylcarbodiimide (DCCD), which is known to inhibit the activity of membrane-bound ATPase (Higuti et al., 1992). Recent study showed that *B. cereus* ATPase activity led to an increase in pH_i_ when cells were exposed to acid stress. Indeed, DCCD had a negative effect on the ability of *B. cereus* cells to survive and maintain their pH_i_ during acid shock. Furthermore, transcriptional analysis revealed that expression of *atpB* (encoding b subunit of F_1_F_0_-ATPase) was increased in acid-adapted cells compared to non-adapted cells before and after acid shock. These data demonstrate that *B. cereus* is able to induce an ATR during growth at low pH, depending on the ATPase activity induction and pH_i_ homeostasis (Senouci-Rezkallah et al., 2015).

##### Decarboxylation of amino acids

The presence of amino acids (glutamate, lysine, and arginine) in food such as vegetables and dairy products can enhance the ability of bacteria to adapt to and survive acid stress. These amino acids can be decarboxylated by systems composed of one or more decarboxylases, which convert their substrates into amine derivatives and carbon dioxide or bicarbonate, and an antiporter that then exchanges each amino acid for its decarboxylated amine. Decarboxylation of amino acids controls bacterial pH by consuming hydrogen through the decarboxylation reaction. The lysine, arginine and GAD systems predominate in acid tolerance (Booth, 2002). The GAD system has been identified in a variety of bacteria such as *E. coli. L. monocytogenes. Shigella flexneri*, and *Lactococcus lactis* (Cotter and Hill, 2003; Cotter et al., 2005; De Biase and Pennacchietti, 2012; Kanjee and Houry, 2013). In *E. coli*, known components of glutamate-dependent acid resistance include two isoforms of GAD (GadA and GadB) and a putative glutamate: γ-aminobutyric acid (GABA) antiporter called GadC. GadA/GadB is assumed to catalyze the conversion of protonated glutamate to GABA, whereas GadC exports GABA in exchange for a new extracellular glutamate molecule. This process consumes protons in cells, which eventually increases pHi, protecting the cell from the damage caused by acid shock (Richard and Foster, 2003). Three decarboxylases (GadD1, GadD2, and GadD3) and two antiporters (GadD1T1 and GadD2T2) were identified in *L. monocytogenes*. The GadD2/T2 system was found to be responsible for the survival of the cells in acidic conditions at pH 2.8. GadD1T1 plays a role in growth at moderately acidic pH values (5.1) (Cotter et al., 2005). Gene encoding GAD was identified in *B. cereus* ATCC 10987, while no gene encoding GABA glutamate exchanger was found. As a result, the *gad* gene was not up-regulated under low-pH exposure (Mols et al., 2007, 2010a,b). Unlike ATCC 10987, *B. cereus* ATCC 14579 genome does not contain *gad* gene. However, glutamate enhanced the resistance of *B. cereus* ATCC 14579 cells to pH 4.0 acid shock (Senouci-Rezkallah et al., 2011).

The system AD system is composed of a cytoplasmic arginine decarboxylase (AdiA) and an arginine/agmatine antiporter (AdiC). After proton-consuming decarboxylation of arginine by AD to give agmatine in the cell, the agmatine is carried out of the cell by the antiporter in exchange for arginine. The consumption of protons during decarboxylation reduces acidity in the cytoplasm. In *E. coli*, the presence of arginine during acid shock raised pH_i_ from 3.7 to 4.7, showing that AD could enable pH_i_ homeostasis during acid stress (Richard and Foster, 2004). In *S. typhimurium*, the AD system is active only under acid growth conditions (Kieboom and Abee, 2006; Alvarez-Ordonez et al., 2010c). In *B. cereus*, two genes have been annotated as encoding ADs [*speA* and *yaaO*, (Ivanova et al., 2003)], but no gene has been annotated as encoding an arginine/agmatine antiporter. However, it has been shown that the presence of arginine improved acid stress resistance of *B. cereus* cells. It is thus probable that *B. cereus* utilizes the AD system for surviving in acidic environment (Senouci-Rezkallah et al., 2011).

The lysine decarboxylase system is composed of a decar-boxylase (CadA) and a lysine/cadaverine antiporter (CadB). After decarboxylation of lysine to cadaverine in the cell by proton-consuming lysine decarboxylase, the cadaverine is carried out of the cell by the antiporter in exchange for lysine. The consumption of protons during the decarboxylation reaction again lowers acidity in the cytoplasm, allowing the homeostasis of pH_i_ during acid shock. In *E. coli* and *Vibrio vulnificus*, the lysine decarboxylase system is encoded by the *cadBA* operon. This operon is activated by CadC, and repressed by LysP (Neely and Olson, 1996; Rhee et al., 2005). In *Vibrio parahaemolyticus*, the expression of *cadBA* has been shown to increase in the presence of lysine. The mutation of the gene *cadA* also impaired survival in acid shock conditions relative to the wild-type strain, showing the role of this system in resistance to acid shock by maintenance of pH_i_ (Tanaka et al., 2008). In *B. cereus* ATCC 14579, the gene encoding the enzyme lysine decarboxylase is *yvdD* (Ivanova et al., 2003), but the gene encoding the antiporter has not yet been identified. Like glutamate and arginine, the addition of lysine improves *B. cereus* resistance to acid stress, suggesting a role of the lysine decarboxylase system in this bacterium (Senouci-Rezkallah et al., 2011).

##### Buffering ability of cytoplasm

Independently of the involvement of the decarboxylation systems, the pHi of a cell can be stabilized by the relatively high buffering ability of the cytoplasm. When acid or alkaline compounds enter the cell, the buffering action of the cytoplasm tends to offset these variations and keep the pHi neutral. Rius and Loren (1998) compared the buffering ability of *Bacillus alcalophilus*, an alkaliphilic bacterium, with those of *B. subtilis* and *S. aureus*, both neutrophilic bacteria. They showed that the buffering ability of *B. alcalophilus* was influenced by the culture pH and other conditions. In addition, this buffering ability was greater in anaerobic growth conditions than in aerobic growth conditions, clearly showing that the cells have a significantly greater buffering activity in a fermenting medium. In *B. cereus*, a study showed that cells grown in unregulated batches at pH_e_ 7.0 showed a pH_i_ of 9.0, which fell to 7.9 and 6.2 when the cell growth at pH 7.0 was followed by 1 h incubation at pH 6.3 and 4.6, respectively. It has also been observed that the pH_i_ of cells adapted at pH 6.3 was higher (pH_i_ 6.6) than that of non-adapted cells (pH_i_ 6.1) (Browne and Dowds, 2002). However, in unregulated batch culture, the cell environment is not controlled and several factors, such as growth phase, growth rate, and carbon and oxygen resource availability can therefore also influence pH_i_.

#### Cell Density

In addition to responses to several stresses, bacteria are known to regulate diverse physiological processes in a cell density-dependent manner. Cell density-dependent regulation appears to follow a common theme, in which a small, self-generated molecule is exported as the signal for intercellular communication, commonly called quorum sensing. Cell density was found to modulate acid adaptation in *Streptococcus mutans* log-phase cells, since pre-adapted cells at a higher cell density or from a dense biofilm displayed significantly higher resistance to the killing pH than the cells at a lower cell density (Li et al., 2001). The authors also showed that mutants defective in the *comC. comD*, or *comE* genes, which encode a quorum sensing system essential for cell density-dependent induction of genetic competence, had a diminished log-phase ATR. They concluded that optimal development of acid adaptation in *S. mutans* involves both low pH induction and cell–cell communication. Also in this strain, the gene *luxS* involved in the signal synthesis in quorum sensing plays a role in the regulation of tolerance to acid stress (Wen and Burne, 2004). The synthesis of LuxS is induced in *E. coli* on exposure to acetic acid, suggesting that its expression in this organism is induced at low pH (Frees et al., 2003b). Four quorum sensing systems operate in *B. cereus* and, one of them controls the oxidative stress response (Slamti et al., 2014). Exposure to acid stress induces secondary oxidative stress in *B. cereus* (Mols and Abee, 2011a). Therefore, the quorum sensing system that controls oxidative stress could be involved in acid adaptation.

### Protection and Repair of Proteins and DNA

In an acid stress situation, proteins can undergo modifications, from changes in conformation to complete denaturing, all of which will significantly affect their activity. Denatured proteins are dealt with chaperone proteins, such as DnaK/DnaJ and GroES/EL (Susin et al., 2006). In *L. lactis*, these chaperones are induced at pH 4.5, and not at pH 5.5, and therefore respond at a certain level of acidity in the medium, or a certain concentration of denatured proteins in the cytoplasm (Frees et al., 2003b). In *S. mutans*, the expression of *dnaK* and the quantity of DnaK are higher in acid-adapted cells than in non-adapted cells, and increase in response to an acid shock (Jayaraman et al., 1997), suggesting that the regulation of *dnaK* by pH is transcriptional in this bacterium. In *S. typhimurium* DT104, an increase level of DnaK and GroEL was observed in acid-adapted cells (Berk et al., 2005). Exposure of *L. lactis* to low pH revealed that, in addition to DnaK and GroEL, several heat shock proteins are part of the acid shock response. Among them are ClpE and ClpP (Frees et al., 2003a). The Clp proteins are ATPase-dependent proteases involved in the turn-over of denatured proteins: they are responsible for the rapid degradation of damaged proteins and the regulation of the levels of some proteins in the cell (enzymes and regulators). In *B. cereus*, DnaK is overproduced in response to acid shock (Browne and Dowds, 2002). Proteins of 66 and 59 kDa, which could be, respectively, DnaK and GroEL, have also been found expressed in stationary phase cells irrespective of pH (Jobin et al., 2002). More recent study showed that chaperone-encoding genes *dnaK* and *groES* and protease-encoding gene *clpC* were up-regulated upon exposure to sublethal acid shocks (Mols et al., 2010b), suggesting that these proteins are involved in acid stress response in this bacterium.

When pH_i_ becomes too acidic, DNA loses purine and pyrimidine units (Cotter and Hill, 2003). First the bases are protonated and then, the glucoside bonds are cleaved. The ultraviolet (UV) excinulease system UvrA-UvrB which is known to repair damage caused to DNA by UV radiation or exposure to many chemical agents (Lage et al., 2003) is associated with low-pH adaptation. The UvrA–UvrB complex identifies the changes in conformation or structure in the damaged DNA. UvrA then dissociates from UvrB, which remains bound to the DNA. UvrC in turn binds to the UvrB-ADN complex and effects the incision of seven nucleotides. UvrD intervenes to remove UvrC and the damaged nucleotides. UvrB remains bound to the DNA until DNA polymerase I synthesizes the excised sequence (Skorvaga et al., 2004). In *S. mutans. uvrA* is induced by acidity. In addition, a Δ*uvrA* mutant is more sensitive to growth at pH_e_ 5.0 than the wild-type strain, and when it is pre-incubated at a non-lethal acidic pH, it is unable to survive at pH 3.0. This indicates that UvrA is involved in adaptive response to low pH (Hanna et al., 2001). In *Methylobacterium dichloromethanicum*, a Δ*uvrA* mutation significantly limited viability and growth on dichloromethane, when intracellular hydrochloric acid is produced. This dehalogenation produced a genotoxic intermediate compound. However, it is not yet known whether DNA lesions are caused by this reaction product or by the acidity (Kayser et al., 2002). Mutations in the genes *recA* (coding for a protein involved in homologous recombination and which is a regulator of the SOS response) and *uvrB* caused a significant decrease in tolerance to low pH in *H. pylori* (Thompson and Blaser, 1995; Thompson et al., 1998), showing the importance of these DNA repair mechanisms for survival in acid conditions. In *B. cereus*, the *uvrA* and *uvrB* genes are up-regulated upon exposure to pH 5.5 set with lactic acid, suggesting a role of the UvrA–UvrB system in stress acid resistance (Mols et al., 2010b).

### Concluding Remarks

Although much has been learned about other bacteria respond to acid stresses, there remains a great deal to discover, making *B. cereus* acid stress responses a fruitful and important area of future research. In addition, the majority of studies that have analyzed the response of food-borne pathogens to acid stress were performed in presence of oxygen. Anoxia may thus provide attractive condition to study the acid resistance mechanisms in *B. cereus* and other food-borne pathogens.

## Oxygen Sensing and Adaptation to Anoxia

*Bacillus cereus* is a facultative anaerobic microorganism, i.e., it can survive at various levels of oxygenation. The common terminology to describe the various oxygen conditions, such encountered by *B. cereus* in food and GI tract is based on a comparison with the atmospheric level, which is 21% (v/v), a level referred to as normoxia. Hypoxia is a condition where oxygen concentration is lower than 21%. No free oxygen is available under anoxia.

### *B. cereus* Central Metabolism and Redox Homeostasis

Whatever the oxygenation conditions, *B. cereus* catabolizes glucose through glycolysis and, to a lesser extend, through the pentose phosphate pathway (PPP; Zigha et al., 2006). Glycolysis and PPP are fuelled by the phosphoenolpyruvate (PEP)-dependent phosphotransferase system (PTS), a transport system that cells use to bring glucose into the cytoplasm using energy transferred by PEP (Deutscher et al., 2006; Cao et al., 2011; Warda et al., 2016). Glycolysis produces two molecules of pyruvate per molecule of activated glucose (glucose-6-phosphate, G-6P) and, in the process, reduces two molecules of NAD^+^ to NADH and produces a net gain of two ATP molecules (**Figure 2**). In addition to providing ATP by substrate-level phosphorylation, glycolysis is a major source of metabolic intermediates for biosynthetic pathways. The processing of activated glucose (G-6P) through the PPP also produces biosynthetic precursors. Two of these biosynthetic precursors, ribose-5-phosphate, and erythrose-4-phosphate, are essential for the synthesis of nucleotides, histidine, and aromatic amino acids. In addition to providing biosynthetic intermediates, the PPP generates two molecules of NADPH per molecule of G-6P. NADPH provides the reducing power that drives numerous anabolic reactions, including those responsible for the biosynthesis of all major cell components (Spaans et al., 2015). NADPH is also required to maintain and regenerate the cellular detoxifying and anti-oxidative defense systems (Agledal et al., 2010). The antioxidant defense system of *B. cereus* is constituted by an elaborate, often overlapping network of enzymes, such as superoxide dismutase (SOD), catalase, flavohemoglobin, peroxiredoxins, thioredoxins, among others, and low molecular mass (LMW) thiols such bacillithiol (BSH), coenzyme A (CoASH) and cysteines (Newton et al., 1996; Fang et al., 2013). In addition to their roles in detoxification of ROS, LMW thiols, which are present in millimolar concentrations in the cytoplasm, function as major thiol-redox buffers to maintain the redox state of the cytoplasm (Newton et al., 2009; Loi et al., 2015). The PPP and glycolysis are linked by transketolase and transaldolase that convert ribose-5-phosphate into glyceraldehyde-3-phosphate and fructose-6-phosphate. Fructose-6-phosphate is a precursor for *N*-acetylglucosamine, which is required for BSH (Helmann, 2011). Increased carbon flow through the PPP is often associated with stressful conditions or infections in Gram-positive pathogens (Eisenreich et al., 2006; Bergman et al., 2007).

In the presence of oxygen, pyruvate is converted to acetyl coenzyme A (Acetyl-CoA) by the pyruvate dehydrogenase complex (Pdh) (Duport et al., 2006). Acetyl-CoA can enter into the tricarboxylic acid cycle (TCA) via a condensation reaction with oxaloacetate that is catalyzed by citrate synthase. Then two carbons are lost as CO_2_ for every two carbons (i.e., Acetyl-CoA) that enter the TCA. TCA provides three biosynthetic intermediates that are critical for the *novo* synthesis of many amino acids and porphyrins: oxaloacetate, α-ketoglutarate, and succinate/succinyl-CoA. The reducing equivalents generated by the glycolysis and TCA (NADH and FADH) are reoxidized through the aerobic respiratory chain, resulting in the build-up of a proton motrice force and the subsequent synthesis of ATP by ATP synthase (38 ATP per molecule of consumed glucose). Although it has not been thoroughly studied, the aerobic respiratory chain of *B. cereus* resembles the respiratory chain of *B. subtilis* (Contreras-Zentella et al., 2003; Rosenfeld et al., 2005; Garcia et al., 2008; Melo and Teixeira, 2016). It contains two major branches, one quinol oxidase branch (with cytochrome *bd* or cytochrome *aa3* as its terminal oxidase) and one cytochrome oxidase branch (with cytochrome *caa3* as its terminal oxidase) (**Figure 2**). The terminal oxidases catalyze the four-electron reduction of dioxygen to two water molecules. The aerobic respiratory chain is not only a main source of ATP but it also a major source of reactive oxygen species (ROS; Gonzalez-Flecha and Demple, 1995; Messner and Imlay, 2002; Mailloux et al., 2011). ROS generation starts with the formation of a superoxide anion (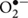). Within the respiratory chain, NADH: menaquinone oxidoreductase (complex I) and *bc* complex (complex III) are generally considered as the main producers of 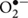 (**Figure 2**). Dismutation of 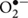 (either spontaneously or through a reaction catalyzed by SODs) produces hydrogen peroxide (H_2_O_2_), which in turn may be fully reduced to water or partially reduced to hydroxyl radical (OH^∙^), one of the strongest oxidants in nature (Imlay, 2013). ROS production serves as a metabolic signal and under normal conditions are quenched by the antioxidant defense system to maintain them to non-toxic levels. However, when released in excess under certain stress conditions such as hypoxia and change in pH that abruptly affect the electron transport chain, ROS can also directly damage cells (Mols et al., 2009, 2010b, 2011).

When the oxygen concentration drops to a level at which oxygen becomes limiting as a substrate for cytochrome *c* oxidase, ATP production via oxidative phosphorylation is no longer able to meet cellular demands for ATP. This can be compensated by the activation of glycolytic activity to increase ATP production by substrate-level phosphorylation. However, compared with oxidative phosphorylation, the ATP production through glycolysis alone is much lower. Therefore, glycolytic activity must be strongly up-regulated under hypoxic conditions to generate sufficient ATP. This phenomenon, namely the Pasteur effect requires the efficient recycling of NAD^+^ from NADH, otherwise glycolysis will become limited by the availability of NAD^+^. Therefore, the fermentative pathways are induced by activating the expression of key enzymes.

Under anoxia and in absence of external electron acceptor, *B. cereus* carries out mixed acid-butanediol fermentation (**Figure 2**). Lactic acid is the major by-product of fermentation (more than 60% of total production) both at neutral and acidic pH (Duport et al., 2004, 2006; Messaoudi et al., 2010; Le Lay et al., 2015). The pyruvate-to-lactate pathway involves three L-lactate dehydrogenases, LdhA, B, and C. It has been shown that LdhA exerted a major control on both *B. cereus* fermentative growth and enterotoxin production (Laouami et al., 2011). The conversion of pyruvate to acetyl-CoA and formate requires high levels of pyruvate formate lyase (Pfl) compared to Pdh complex, probably to avoid excessive NADH formation under fermentative conditions (Duport et al., 2006). The acetyl-CoA is converted to acetate through the ATP-producing acetate pathway and to ethanol through the NADH-recycling ethanol pathway. At neutral pH, acetate and formate are produced in similar amounts (each accounting for ∼15% of total production), while ethanol, and to higher extend succinate and 2,3 butanediol are minor fermentation products (Duport et al., 2004; Rosenfeld et al., 2005). However, the relative rate of formation of all these glucose by-products is influenced by the ORP of the growth medium (Zigha et al., 2006). At acidic pH, production of 2,3-butanediol highly increased at the expense of acetic acid and succinic acid and to lesser extend lactic acid (Le Lay et al., 2015). During fermentative growth, TCA functions only to supply biosynthetic precursors and is transformed from a cyclic pathway to two oppositely oriented half cycles (**Figure 2**). In fermenting cells, the direct formation of ROS is abolished by the absence of oxygen. However, several anoxia-specific alterations can promote the oxidative response (Lumppio et al., 2001; Rusnak et al., 2002). The occurrence of an oxidative component in response to oxygen deprivation has been confirmed in *B. cereus* by microarray studies on the whole genome level and by proteomic studies (Mols and Abee, 2011b; Clair et al., 2012; Madeira et al., 2015).

Under anoxia, *B. cereus* can growth via nitrate ammonification (Zigha et al., 2006). Nitrate in the human intestine originates both from endogenous synthesis and dietary products rich in nitrate (Tannenbaum et al., 1978; Lidder and Webb, 2013). During nitrate respiration, nitrate is reduced by the respiratory nitrate reductase (NarGHI) to nitrite in *B. cereus* cells. Nitrite is further reduced to ammonia by a general nitrite reductase (NasDE). Nitrate reduction is coupled to ATP generation through proton motrice force (Rosenfeld et al., 2005). Due to the drastically different ATP yields of respiratory and fermentative processes, *B. cereus* uses a fine-tuned regulatory system to maintain the most efficient mode of ATP generation under anoxia (Zigha et al., 2006). Nitrate-respiring *B. cereus* cells produce nitric oxide (NO) as an intermediate product of nitrate reduction to N_2_O (Kalkowski and Conrad, 1991). The chemical properties of NO make this gas a good candidate for a signaling molecule (Gusarov and Nudler, 2012). Gram-positive bacteria, including *B. cereus* can also generate NO through a bacterial analog of mammalian NO synthase (bNOS) in presence of oxygen. It has been shown that bNOS from *B. subtilis. B. anthracis* displayed NO-forming activity dependent on arginine (Adak et al., 2002; Gusarov et al., 2008). The bNOS-mediated NO was implicated in the protection of bacteria against oxidative stress, a variety of antibiotics and other stresses such as acid stress (Tan et al., 2010; Gusarov and Nudler, 2012). NO also regulates growth and pathogenicity of *B. anthracis* (Popova et al., 2015).

### Exoproteins and Virulence Factors Supporting Pathogenesis

*Bacillus cereus* excretes high level of proteins into the extracellular medium. However, the level of excreted proteins is lower during anaerobic fermentative growth than under aerobic respiratory growth (Madeira et al., 2016a,b). Like transcription and translation, exportation of proteins is an energetically expensive process. Therefore, the decrease of protein excretion in fermentative cells may be attributed to decreased energy availability. Most exoproteins are secreted as precursors with a cleavable N-terminal signal sequence, but a significant fraction is secreted by non-classical pathways, i.e., without signaling peptides and sequence motifs for surface anchoring. Six signal peptide-dependent pathways are currently recognized in Gram-positive bacteria (Schneewind and Missiakas, 2014): the general secretory (Sec) pathway, the twin arginine targeting (Tat) pathway, the fimbrillin-protein exporter, the flagellar export apparatus, the holins, and the ESAT-6/WXG100 secretion system. The Sec pathway is considered the general housekeeping protein translocation system and is essential in *B. cereus* (Fagerlund et al., 2010; Senesi and Ghelardi, 2010; Senesi et al., 2010). The proteins arising from cellular secretion and other protein export mechanisms are components of the *B. cereus* exoproteome (Armengaud et al., 2012). *B. cereus* exoproteome has been the focus of several shotgun proteomic studies (Clair et al., 2010, 2013; Laouami et al., 2014; Madeira et al., 2015, 2016b). These proteomic studies identified up to 377 different exoproteins. Among them 65 putative virulence factors were identified, including 15 toxin-related proteins, 12 motility-related proteins and 36 adhesins and degradative enzymes (Madeira et al., 2015, 2016a,b). These virulence-related proteins represent more than 85% of exoproteins whatever the growth condition (Clair et al., 2010; Madeira et al., 2015). *B. cereus* exoproteome includes numerous cytoplasmic proteins involved in metabolic pathways (mainly glycolysis) and oxidative stress response. Many of these proteins are conserved in the exoproteome of pathogens. A significant number of these extracellular cytoplasmic proteins have been found to serve two or more functions and are referred as “moonlighting” proteins (Henderson and Martin, 2011; Gotz et al., 2015). Moonlight proteins have been shown to localize at the cell surface and participate in adhesion, colonization and virulence (Henderson and Martin, 2011; Ebner et al., 2016a,b). Surface-associated moonlight proteins have been reported to be reversible and pH dependent (Nelson et al., 2001; Antikainen et al., 2007). Extracellular cytoplasmic proteins are mainly excreted during the stationary growth phase (Yang et al., 2011) and in *B. cereus*, their time dynamic is negatively correlated to the dynamic of toxin-related proteins, indicating that a specific selection process has to occur (Madeira et al., 2015).

The toxin-related proteins found in *B. cereus* exoproteome include the lytic components (L1 and L2) and the binding component (B) of Hemolysin BL (Hbl). Hbl requires all three components for full activity (Beecher et al., 1995). Hbl may form a pore similar to other soluble channel-forming proteins in host cell membranes (Madegowda et al., 2008; Stenfors Arnesen et al., 2008). The tripartite Hbl complex is encoded by genes clustered into a polycistronic operon with the transcriptional order *hblC. hblD*, and *hblA* (Ryan et al., 1997). An ORF, name *hblB*, is located immediately downstream of *hblCDA* in the *B. cereus* ATCC 14579 genome and is transcribed independently (Clair et al., 2010). *hblB* encodes HblB’, which is structurally related to the B component of the Hbl complex. Its activity is currently unknown (Clair et al., 2010). NheA, NheB, and NheC are the three components of the non-hemolytic enterotoxin (Nhe) and are encoded by the *nheABC* operon (Lindback et al., 2004). All three components NheA, NheB, and NheC are required for full toxic activity, although NheC is only expressed in small amounts due to translational repression (Lindback et al., 2004). Nhe is a pore-forming toxin (Didier et al., 2012; Phung et al., 2012). Cytotoxin K (CytK) is a single-component protein toxin and belongs to the family of β-barrel pore-forming toxins (Baida et al., 1999; Lund et al., 2000). CytK possesses dermonecrotic, cytotoxic, and hemolytic activities (Lund et al., 2000; Hardy et al., 2001). HlyI is a thiol-activated cholesterol-binding cytolysin (Kreft et al., 1983; Minnaard et al., 2001; Ramarao and Sanchis, 2013). All the genes encoding Hbl, Nhe, CytK, and HlyI belong to the PlcR virulence regulon (Gohar et al., 2008). Hemolysin II (HlyII) is cytotoxic due to its ability to disrupt cellular and artificial membranes by pore formation (Andreeva et al., 2006). EntFM exhibits three protein–protein interaction SH3 domains and a NlpC/P60 domain that shares similarities with cell wall peptidase. There is controversy over the role of EntFM in *B. cereus* cytotoxicity, but wide consensus on its role in pathogenicity (Boonchai et al., 2008; Tran et al., 2010). The structurally related proteins EntA, EntB, EntC, and EntD were annotated as “enterotoxin/cell wall-binding proteins” because they possess, in addition to two SH3 domains, an extracellular cell wall-binding 3D domain (Clair et al., 2010). Recent study showed that EntD plays a crucial role in maintaining cell wall structure and that, in the absence of EntD, *B. cereus* cells are able to reoriente their metabolism to maintain cell wall integrity. Such adaptation program leads to decreased virulence factor production, specifically Nhe and Hbl production (Omer et al., 2015). The functions of EntA, EntB and EntC are currently unknown.

Taken together, the 15 toxin-related proteins found in *B. cereus* exoproteome represent more than 30% of exoproteins during the exponential growth phase, suggesting that they may have an important cellular function for the producer bacterium (Clair et al., 2010). Toxin-related proteins contain methionine residues that are susceptible to intracellular oxidation in both respiratory and fermenting cells (Madeira et al., 2015). Methionine residues of proteins are known to act as ROS scavengers (Luo and Levine, 2009). High level secretion of toxin-related protein during active growth may thus contribute to the protection of *B. cereus* cells against cellular oxidation and maintain redox homeostasis by keeping endogenous ROS at bay whatever the oxygen condition.

The regulation of *B. cereus* toxin gene expression mobilizes a complex machinery (Ceuppens et al., 2011; Jessberger et al., 2015) that includes the virulence regulator PlcR (Salamitou et al., 2000; Gohar et al., 2008) and several transcriptional regulators that coordinately control metabolic and virulence genes such as the CodY repressor (Lindback et al., 2012; Bohm et al., 2016), the ferric uptake regulator Fur (Sineva et al., 2012), the catabolite control protein A [CcpA, (Van der Voort et al., 2008)] and the redox regulators, Fnr, ResD, Rex, and OhrR. All these four redox regulators are able to regulate directly the expression of *hbl* and *nhe* after binding to the promoter region of *hblCDA* and *nheABC* (Esbelin et al., 2008, 2009; Clair et al., 2013; Laouami et al., 2014). In addition, it was shown that ResD and Fnr form a ternary complex with the virulence regulator PlcR (Esbelin et al., 2012).

### Redox Regulators that Coordinate Central Metabolism and Production of Toxin-Related Proteins

**FIGURE 3:**
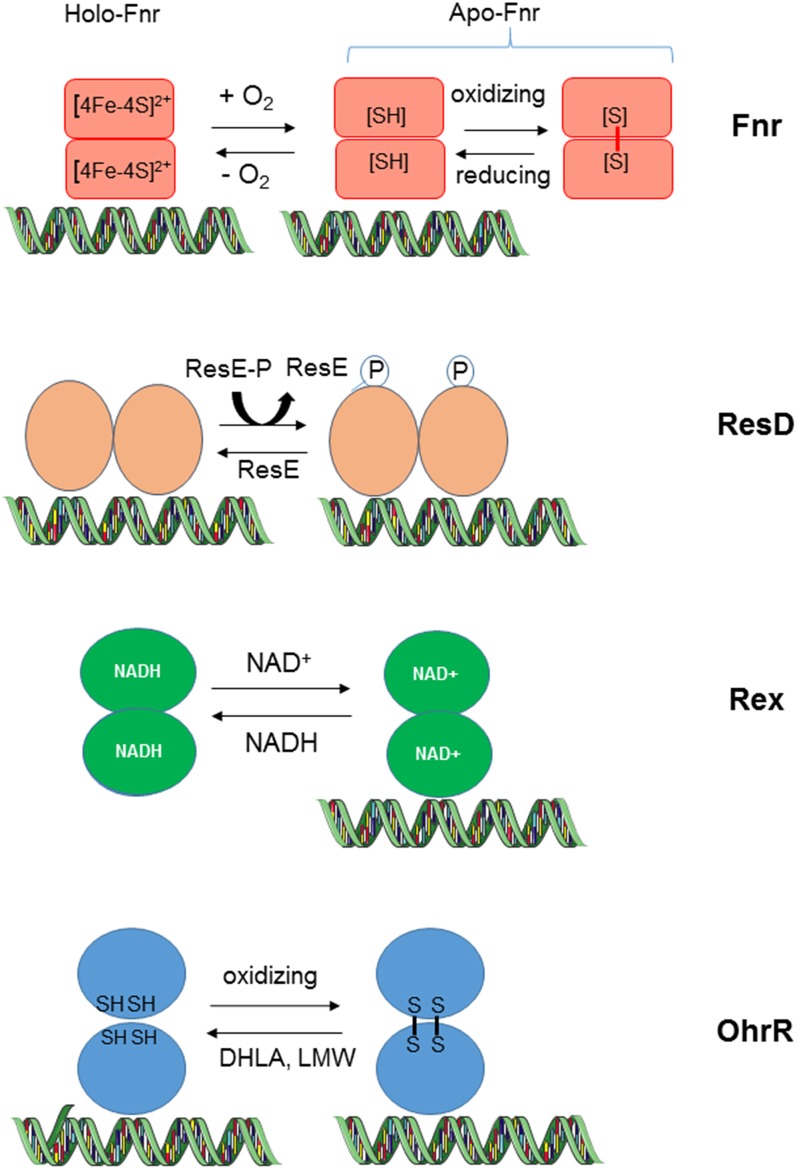
**Redox-sensing mechanisms of transcriptional regulators in *B. cereus*.** HoloFnr binds one [4Fe-4S]^2+^ cluster per monomer. This cluster is degraded upon exposition to oxygen. ApoFnr (clusterless) is active as a DNA binding protein under its reduced dimeric form. The oxidized ApoFnr form, which is stabilized by means of one or more SS bonds, is inactive. The thiol-based redox switch mediates a response to oxygen concentration or cellular oxidant (see text). Binding of ResD to DNA is not dependent on ResD phosphorylation status, which is regulated by the kinase and phosphatase activities of ResE. Rex senses the intracellular redox status through changes in the NADH/NAD^+^ ratio. Unlike the NAD^+^-bound Rex form, the NADH-bound Rex form is incapable of binding DNA. OhrR senses oxygen concentration or ROS through cysteine residues (see text). OhrR is a non-covalent dimer in its reduced form and a covalent dimer in its oxidized form. Dihydrolipoate (DHLA) and low molecular mass thiols (LMW) participate in the recovery of reduced OhrR from the oxidized form. *B. cereus* OhrR can bind DNA both under its reduced and oxidized form.

A number of classical sensors/regulators are employed by various species of bacteria to sense oxygen changing conditions (Bueno et al., 2012). These sensors/regulators include Fnr, ResDE, Rex, and OhrR (**Figure 3**). All of them could orchestrate the expression of virulence determinants in *B. cereus*, both directly, and indirectly, by impacting key metabolic and regulatory circuits.

#### B. cereus Fnr

The *B. cereus* Fumarate and nitrate reductase regulator Fnr is a member of the Crp/Fnr (cyclic AMP-binding protein/fumarate nitrate reduction regulatory protein) family of helix-turn-helix transcriptional regulators (Korner et al., 2003). Like all the members of the Crp/Fnr family, *B. cereus* Fnr contains an N-terminal region made up of antiparallel β-strands able to accommodate a nucleotide, and a C-terminal extension with four cysteine residues that coordinate a [4Fe-4S]^2+^ cluster (Esbelin et al., 2009). Fnr is essentially present in the apo-form (clusterless) in aerobically grown cells, and in the holo-form in anaerobically grown cells (Esbelin et al., 2012). Under aerobiosis, Fnr is able to sense oxygen concentration changing and probably ROS and NO (Jiang et al., 2016) through the oxidation of Cys thiol groups, which link two monomers in an inactive dimer. The inactivation of Fnr by oxygen is reversible. In the current model for Fnr function, the active dimeric forms of Fnr bind to the promoter region of target operons/genes to activate or repress their transcription. Fnr plays a key role within the regulatory cascade governing fermentative pathways in *B. cereus* because (i) its transcription is strongly induced in fermenting cells and (ii) the inactivation of its gene abolishes fermentative growth. The role of Fnr under anaerobic and aerobic respiratory growth is more moderated (Zigha et al., 2007).

#### B. cereus ResDE

*The* ResDE two-component signal transduction system consists of a membrane-bound histidine sensor kinase (ResE) and a cytoplasmic response regulator (ResD). The *resD* and *resE* genes compose a transcriptional unit included into a larger operon that comprises *resABC;* these three genes encode proteins similar to those involved in cytochrome *c* biogenesis (Duport et al., 2006). The *B. cereus resABCDE* locus is organized similarly to that in *B. subtilis* and *B. anthracis* (Sun et al., 1996; Wilson et al., 2008). The ResDE two-component system regulates the expression of several genes of the fermentative and respiratory pathways in *B. cereus*. However, it appeared to exert a more important role in anaerobic fermentative pathways (with a more pronounced effect under high reductive conditions) than in aerobic respiratory pathways. Unlike *fnr*, the *resDE* mutation did not abolish the fermentative growth of *B. cereus*, indicating that although it plays an important role, it is not indispensable for *B. cereus*. The ResDE system is modulated primarily by the autophosphorylation activity of ResE at a conserved histidine residue. The redox signal activating ResE has not been identified in *B. cereus* but have been postulated to be the redox state of menaquinones in *B. subtilis* under aerobiosis (Geng et al., 2007). Upon activation, ResE donates a phosphate to its cognate regulator, ResD. The phosphatase activity of ResE controls the level of phosphorylated ResD (ResD∼P). In *Bacillus subtilis*, phosphatase activity of ResE is regulated by oxygen availability and anaerobic induction of the ResDE regulon is partly due to a reduction of the ResE phosphatase activity during anaerobiosis (Nakano and Zhu, 2001). ResE is the only relevant kinase able to phosphorylate ResD. However, it was proposed that acetyl-phosphate (produced through the acetate pathway) could transfer its phosphate to ResD (Gueriri et al., 2008), linking its phosphorylation state to the metabolic status of the cell. Both phosphorylated and unphosphorylated forms of dimeric *B. cereus* ResD are able to bind DNA but their DNA binding affinity depends on promoter architecture of the target genes. For example, phosphorylation of ResD, which is higher under anaerobiosis than under aerobiosis enhances its ability to bind to its own promoter and *fnr* promoter but not to the enterotoxin gene promoters. Both ResD and ResD∼P physically interacts with Fnr and simultaneously bind their target promoter. A model was proposed on which ResD∼P may act as a Fnr co-activator and ResD as a Fnr anti-activator (Esbelin et al., 2012).

#### *B. cereus* Rex

Changes in oxygen availability and ORP influence the relative level of dinucleotides NAD^+^ and NADH in the cells, and such changes are sensed by the transcriptional regulator Rex. The crystal structure of Rex from *Thermus aquaticus. Thermus thermophilus* in complex with NADH and of *B. subtilis* Rex without cofactor has been determined (Sickmier et al., 2005; Wang et al., 2008). Rex is composed of two structural domains, an N-terminal domain that adopts a winged helix-turn-helix fold that most likely interacts with DNA, and a C-terminal NADH binding domain. In the complex with NADH, the N-terminal domains pack close to each other in a compact dimer. This conformation of Rex is unable to bind DNA. Rex is thus active as a repressor only when the NAD^+^/NADH ratio indicates adequate NAD^+^. By monitoring the NAD^+^/NADH ratio, Rex helps the cells to regulate pathways that regenerate NAD^+^. Typically, *B. cereus* Rex regulates the carbon flow distribution at the pyruvate node by favoring and limiting the carbon flow entry into the NADH-recycling lactate pathway under anoxic and oxic conditions, respectively (Laouami et al., 2014). By controlling this carbon flow, Rex also controls the availability of glycolytic intermediates for macromolecular synthesis as well as supporting NADPH production through different enzymes located in the TCA cycle, glycolysis and PPP (Laouami et al., 2014). In addition to fine-tune the levels of the prooxidant NADH and the antioxidant NADP, Rex regulates directly the synthesis of antioxidant systems like the OhrRA system (Laouami et al., 2014). Rex also regulates directly the expression of *fnr. resD*, and *ldhA*. LdhA activity is a critical factor in *B. cereus* virulence (Laouami et al., 2011).

#### *B. cereus* OhrRA

The *B. cereus* OhrRA (organic hydroperoxide resistance) system comprises a thiol-dependent peroxidase protein (OhrA), which functions as a low ORP sensor under anoxic conditions and a redox-sensing transcriptional regulator (OhrR), which belongs to the MarR family of winged helix-turn-helix DNA binding protein (Panmanee et al., 2006; Dubbs and Mongkolsuk, 2007). The genes encoding OhrR and OhrA form a bicistronic transcriptional unit (Clair et al., 2012). OhrA is usually reported as a protein that detoxifies the organic hydroperoxides (OHP). OHP could result from oxidation of unsaturated FA by molecular oxygen or ROS (Mongkolsuk et al., 1998). This may explain why OhrA is induced under normoxia and low-ORP anoxia where ROS are the by-product of secondary oxidative stress. *B. cereus* OhrR is an atypical OhrR protein because it contains four cysteine residues at its N-terminal domain while most OhrR regulators contain two cysteines. Like its orthologs in other bacteria, *B. cereus* OhrR functions as a transcriptional regulator that binds to its own promoter under its dimeric reduced form. However, unlike most of its orthologs, it may function as a repressor and an activator of several metabolism-related genes. OhrR is mainly a non-covalent dimer in its reduced form and a covalent dimer in its oxidized form. Dihydrolipoate and LMW participate in the recovery of reduced OhrR from the oxidized form and thus may control the activity of the redox-sensing OhrR regulator (Clair et al., 2013). Besides to regulate the antioxidant system, which includes OhrA, *B. cereus* OhrR controls the abundance level of key enzymes of central metabolism. In this way, (i) it modulates the glycolytic flux and restricts *B. cereus* growth under low ORP fermentative conditions and (ii) it sustains high TCA capacity and limits energy spilling through an overflow metabolism under aerobic respiratory growth.

#### Interactions among the Redox Regulators

**FIGURE 4:**
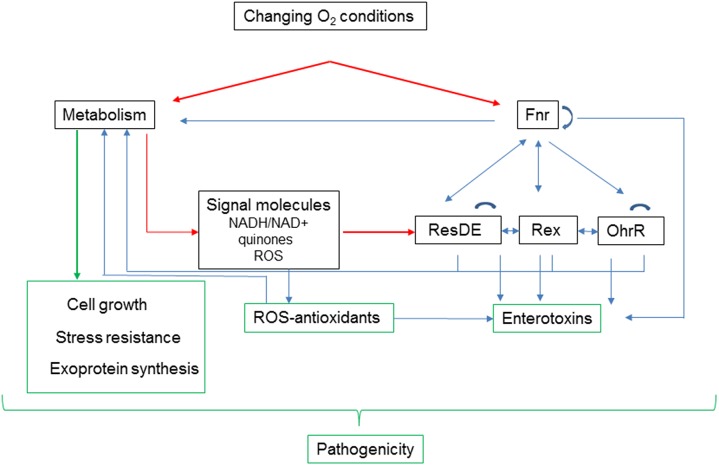
**Adaptation to changing oxygen availability affects *B. cereus* pathogenicity at different levels.** Oxygen acts directly on activity of Fnr and metabolism. Fnr, ResDE, Rex, and OhrR belong to a redox signaling pathway, which interconnects metabolism with expression of enterotoxins. In addition to its role in removing metabolism-generated ROS, antioxidant status modulates the oxidation status of Met residues in enterotoxins (Madeira et al., 2015). Metabolism also contributes directly to pathogenicity by supporting growth and stress resistance, and by modulating exoprotein synthesis.

Although each of the global redox sensors senses different signals, they interact with each other and with operon-specific promoters. Transcriptional activation by Fnr is probably the first response to changing oxygen availability. Fnr regulates its own transcription and the transcription of *resDE* and *rex*, thereby making ResD and Rex more active/inactive as regulators of both catabolic pathways that supply the metabolic intermediates necessary to synthetize enterotoxins, and enterotoxin gene expression. Because Fnr and ResD interact with each other, the change of ResD level generated by Fnr accentuates or reduces this interaction. In addition, the changes of menaquinone redox state and NAD^+^/NADH ratio induced by changing oxygen concentrations also affect the activities of ResD and Rex, respectively; this impacts the expression of *fnr* and their own expression. By regulating the synthesis of the OhrRA system, which is indirectly stimulated by ROS under both normoxia and low-ORP anoxia, Rex also impacts indirectly carbon flow, and enterotoxin synthesis. In conclusion, the regulatory network involving redox sensors is undoubtedly complex but permits the microorganism to coordinate efficiently its central metabolism with enterotoxin production (**Figure 4**).

### Concluding Remarks

Maintaining an appropriate redox balance is essential for *B. cereus* adaptation to changing oxygen availability, and probably for resistance to acid stress (Liu et al., 2016). Redox homeostasis depends of the antioxidant system, which enables bacterial cells to maintain proteins and other cellular components in active state for metabolism. However, to date, we lack knowledge on the intracellular *B. cereus* environment, the behavior of redox couples under different environmental conditions, and the mechanisms of sustained redox homeostasis in *B. cereus*. In particular, a fundamental challenge is to understand how antioxidant-oxidant interactions modulate *B. cereus* pathogenesis.

## Conclusion

*Bacillus cereus* adaptation to acid and low oxygen environments follows pathways that look quite similar to those that have been examined and described in great detail for other bacteria. However, there are also several clear differences that warrant the further examination of the adaptation mechanisms of *B. cereus* to its challenging environments from both a fundamental and industrial point of view.

What is clearly lagging in *B. cereus* research is knowledge of the possible roles that small RNAs (sRNAs) may play in stress responses. Indeed, it is now evident that sRNAs play an essential role in gene regulation under various stress conditions (Babu et al., 2011; Hoe et al., 2013; Miller et al., 2014). Thus, it is crucial that we uncover this important regulatory layer in *B. cereus*, as this will certainly lead to new insights and refine our understanding of the way in which *B. cereus* withstands environmental stress. Another issue that requires attention is the importance of cell individuality in responding to stressors. Cells in a bacterial population, even in a very uniform environment, may differ considerably with respect to the genetic program that is operative under these conditions. Such flexibility occurs in *B. cereus* spores (Van Melis et al., 2014). It would be interesting and important to find out whether culture heterogeneity also plays a role in the responses of *B. cereus* toward acid stress and low oxygen tension (Pandey et al., 2016). Overall, it is expected that investigations of the stress physiology of *B. cereus* will continue to be central to understanding its behavior in challenging environments. Omics approaches available today will lead to important discoveries that can be applied in food safety.

## Author Contributions

CD wrote the paper. MJ and PS contribute to the writing of the part one of the manuscript.

## Conflict of Interest Statement

The authors declare that the research was conducted in the absence of any commercial or financial relationships that could be construed as a potential conflict of interest.
